# Renormalization of the critical exponent for the shear modulus of magnetoactive elastomers

**DOI:** 10.1038/s41598-018-22333-6

**Published:** 2018-03-13

**Authors:** Andrei A. Snarskii, Viktor M. Kalita, Mikhail Shamonin

**Affiliations:** 1 Igor Sikorsky Kyiv Polytechnic Institute, Prospekt Peremohy 37, 03056 Kiev, Ukraine; 2 Institute for Information Recording NAS of Ukraine, Shpaka Street 2, 03113 Kiev, Ukraine; 3 Institute of Physics NAS of Ukraine, Prospekt Nauky 46, 03028 Kiev, Ukraine; 4 East Bavarian Centre for Intelligent Materials (EBACIM), Ostbayerische Technische Hochschule Regensburg, Prüfeninger Strasse 58, 93049 Regensburg, Germany

## Abstract

It is shown that the critical exponent for the effective shear modulus of a composite medium where a compliant polymer matrix is filled with ferromagnetic particles may significantly depend on the external magnetic field. The physical consequence of this dependence is the critical behavior of the relative magnetorheological effect.

## Introduction

In this paper, the renormalization of the critical exponent for the shear modulus of magnetoactive elastomers (MAEs) in an external magnetic field is investigated in two dimensions. An MAE material consists of micrometer-sized magnetizable filler particles dispersed in mechanically soft elastomer matrix^1–8^. The experimentally observed large growth of the shear modulus in magnetoactive elastomers, known as magnetorheological or field-stiffening effect, is still not completely understood from the theoretical point of view^1–8^. In some material realizations, this magnetomechanical effect can be designated as “giant” or even “colossal”, what is expressed by large or very large numbers^9^. It will be shown in the present paper that the change of the critical behavior of the effective shear modulus is caused by the local elastic stresses, arising under the influence of an external magnetic field. This field plays the role of the external parameter renormalizing the critical exponent.

In the narrow sense, the term “renormalization” is usually referred to a particular method in quantum field theory. In a broader sense, this is the appearance of the change in the resulting characteristics of a physical object when interacting with other objects. This broader meaning is considered by us when we investigate such a material property as the shear modulus, more precisely its critical index and the influence of an external magnetic field on it.

The general problem of determining the effective properties of composite materials has been investigated for a long time, the research being commenced probably by James Clerk Maxwell, see, for example, books^10–14^. In the simplest statement of the problem, the task can be formulated in the following way. Consider a composite material comprising two phases with different physical properties. For example, there are rigid spherical inclusions embedded into a soft matrix. The problem consists of finding the effective elastic moduli ***C***^e^ connecting by definition the averaged over the sample’s volume stress tensor 〈***σ***〉 with the strain tensor 〈***ε***〉,

1 $$\langle {\boldsymbol{\sigma }}({\bf{r}})\rangle ={{\bf{C}}}^{{\rm{e}}}\langle {\boldsymbol{\varepsilon }}({\bf{r}})\rangle ,$$

where it is assumed that the following relation is fulfilled locally: ***σ***(***r***) = ***C***(***r***)***ε***(***r***).

Similar problem can be formulated for other physical phenomena, for example, electrical conductivity, dielectric permittivity etc. There is no general solution valid for different types (geometry) of inclusions (spherical, elongated, complex shaped particles, etc.) and their mutual arrangement (micro-structure). For the case of small concentrations of inclusions there exist different approximations, e.g. those of Maxwell, Maxwell Garnett, Bruggeman-Landauer, differential and so on^10–15^.

For the concentrations close to the percolation threshold (when the inclusions form a connected structure, i.e. an infinite cluster), in two-phase composite materials with significantly different physical properties of both phases, a universal behavior of effective coefficients takes place. As it is shown in the percolation theory^10^, effective coefficients (for example, the effective shear modulus ${G}^{{\rm{e}}}(p)$) behave in the vicinity of the percolation threshold as the order parameter in the theory of second-order phase transitions, in particular for *p* < *p*_*c*_

2 $${G}^{{\rm{e}}}(p)=\mu {(1-\frac{p}{{p}_{c}})}^{-{S}_{0}},$$

where *p* is the concentration of rigid inclusions,* p*_*c*_ is the percolation threshold, *S*_0_ is the critical exponent and *μ* is the shear modulus of the matrix.

Critical exponents, *S*_0_, in contrast to the percolation threshold, *p*_*c*_, are universal quantities. This means that their numerical values do not depend on the type of the lattice (if a particular problem is related to lattices) and other details of the internal structure. As in the case of second-order phase transitions critical indices in two and three dimensions are different. Furthermore, they are different for dissimilar physical phenomena, e.g. for conductivity and thermoelectricity.

In the present paper, we will address the basic generalization of the described above calculation procedure for effective coefficients. A particular problem, which will be studied here in detail, is determination of the static shear modulus of MAEs, namely the effect of an external magnetic field on the shear modulus of such a composite material and its critical behavior.

## Effective shear modulus

**Figure 1:**
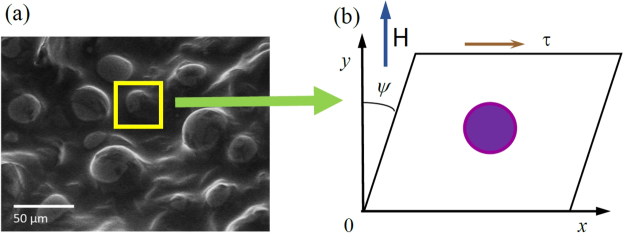
Schematic representation of the shearing experiment in MAEs. (**a**) Real scanning electron microscopy image of spherical ferromagnetic particles in a matrix (^17^ Published by The Royal Society of Chemistry under a Creative Commons Attribution 3.0 Unported Licence). (**b**) Geometry of the shear test.

Recently, a particular mechanism of magnetostriction in MAEs was investigated in^16^. This is the so-called single-particle magnetostriction. An external magnetic field acts on the magnetic moment of a rigid particle surrounded by an elastomer and creates a moment of forces, which in the equilibrium can be compensated only by elastomer’s deformation see Fig. 1.

Figure 1a presents an electron microscopic photograph of an MAE with embedded spherical particles. It serves as an illustration of the internal stricture of a composite material. Figure 1b shows a solitary rigid particle that is rotated and translated as a whole under the engineering shear strain *ψ* caused by the shear stress *τ*. The vector of the magnetic field strength is directed perpendicular to the applied stress: ${\bf{H}}\parallel Oy$.

The mechanism of single-particle magnetoelasticity makes it possible to explain the influence of the magnetic field on the magnitude of the shear modulus in MAEs. The contribution of this mechanism and its influence on the elastic characteristics of the MAE depends on the ratio of the anisotropy constant *K* of the magnetic particle to the shear modulus *μ* of the surrounding elastomer^16^. The greater is the magnitude of the ratio *K/μ*, the stronger is the magnetorheological effect. The magnetic anisotropy constant *K* describes the uniaxial directional dependence of magnetic properties of filler particles^18^. This means that the anisotropy energy *E*_A_ of a single particle can be written in the form of ${E}_{A}=-(KV{\cos }^{2}\theta )/2$, where *θ* is the angle between the easy axis and the particle’s magnetization, *V* is the volume of the particle. The elastomer matrix is non-magnetic and, therefore, its shear modulus *μ* is independent of magnetic field.

The principal difference between the calculation of effective elastic properties of MAEs and the effective elastic properties of nonmagnetic composites is the necessity to take into account the internal degrees of freedom of filler particles, namely directions of the vectors of the magnetic moment of the particles. As a consequence, the magnetic field will cause inhomogeneous elastic stresses in the polymer matrix. The influence of these stresses will inevitably lead to a contribution to the magnitude of the effective shear modulus of the composite material.

In^19^, the equation connecting the shear angle *ψ* and the shear stress *τ* has been obtained. The effective shear modulus *G*^e^, relating the shear angle and the shear stress, has the following form

3 $$\tau ={G}^{{\rm{e}}}(p,H)\psi ,$$

where

4 $${G}^{{\rm{e}}}(p,H)={G}^{{\rm{e}}}(p)+\mu \cdot {\rm{\Omega }}(H)p$$

and

5 $${\rm{\Omega }}(H)=\frac{Hm/K}{(1+Hm/K)(1+4\mu /K)-1}.$$

*p* is the concentration of filler particles, ${G}^{{\rm{e}}}(p)$ is the shear modulus of the composite material in the absence of external magnetic field and *m* is the particle’s magnetization. The case is selected when the directions of easy magnetization axes of all particles coincide with the direction of external field. Therefore, there is no second term in Eq. (3)^19^. Figure 1b illustrates the shear test geometry and the characteristic angles.

The effective modulus ${G}^{{\rm{e}}}(p)$ for non-magnetic composite materials was previously considered and calculated for a large number of different structures and various approximations, see, e.g.^11–14,20^. As a rule, effective coefficients characterizing the properties of composites can be obtained in analytical form only in the approximation of a small concentration of inclusions *p* ≪ 1. From full-scale and numerical experiments, it is known that, in the case of strong inhomogeneity, when the concentration of inclusions approaches the percolation threshold,* p* → *p*_*c*_, the effective coefficients grow strongly. As follows from percolation theory, the behavior of effective coefficients is analogous to the behavior of the order parameter in the theory of phase transitions of the second order and diverges, for example, as the shear modulus in (2) does. The exponent of the power law is called, as in the second-order phase transition, the critical exponent or the critical index.

Calculation of the critical exponent, for example, for the shear modulus of a composite consisting of spherical solid inclusions in an elastic matrix is a complex problem that cannot be accurately analyzed analytically (similarly to the critical exponent of a second-order phase transition). At the same time, critical exponents are crucial characteristics of the behavior of a composite near the percolation threshold and even their approximate calculation is an important statement of the problem.

**Figure 2:**
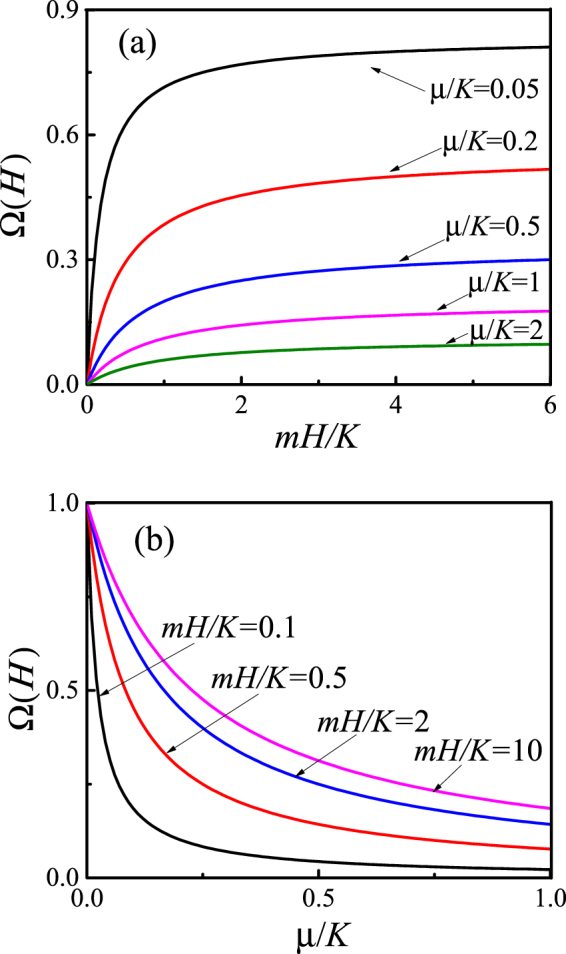
Dependence of Ω(*H*) on the normalized magnetic field *mH*/*K* and magneto-mechanical rigidity *μ*/*K*. (**a**) Dependence of Ω(*H*) on the normalized magnetic field for different values *μ*/*K* = 0.05; 0.2; 0.5; 1; 2. (**b**) Dependence of Ω(*H*) on the magnetomechanical rigidity for different values of the magnetic field *mH*/*K* = 0.1; 0.5; 2; 10.

Figure 2 shows the dependence of the additional term *μ* Ω(*H*) to *G*^e^(*p*,*H*) on the normalized magnetic field *mH*/*K* and the normalized “magneto-mechanical rigidity” of the matrix *μ*/*K*. It is seen that the additional contribution *μ* Ω(*H*) grows with the increasing magnetic field *Hm*/*K* and, in large magnetic fields (*H* > *K*/*m*), comes to the saturation. The magnitude of this magnetoelastic effect is bigger for larger anisotropy constants of the inclusion and smaller shear moduli of the matrix.

In the absence of magnetic field, the effective modulus increases with the growing concentration of inclusions and near the percolation threshold behaves like an order parameter in the theory of second order phase transitions^21–24^, namely, it diverges accordingly to a power law. Qualitatively, this increase is explained by the fact that, as the concentration grows, finite clusters appear and their elastic interaction through the matrix increases. The imposition of an external magnetic field leads to an additional increase in the interaction (additional elastic stresses appear in the matrix). These additional stresses also increase with growing concentration. This effect should lead to the field dependence of the critical index. To estimate the influence of the magnetic field on the critical exponent of the shear modulus, its renormalization, we use the method of Padé approximants^21,25,26^.

## Method of Padé approximants

Calculations of the effective modulus ${G}^{{\rm{e}}}(p,H)$ have been made in the approximation of the small parameter. In our case, this small parameter is the concentration of inclusions *p*. However, by using (4) and (5) one can estimate the behavior of ${G}^{{\rm{e}}}(p,H)$ also at large concentration values in the critical region close to the percolation threshold. For this purpose, we resort to the method of Padé approximants (PA).

The method of Padé approximants is one of the approaches for approximate (and, in some cases, sufficiently accurate) methods of calculating critical exponents for the case when the behavior of effective coefficients at low concentrations is known^25^. The PA method is one of extrapolation methods for estimating the asymptotic behavior of a power series from several first terms of the behavior (see^21^, Chapter 6).

In the theory of second order phase transitions^21–24^, method of Padé approximants was used in the past for solving one of the most difficult problems of statistical physics, namely calculation of critical exponents. As it is written in^21^, this method had a stunning success. In particular, the critical exponent of the magnetization was obtained on the basis of the low-temperature series of magnetization^21^.

The formulation of the problem of determining the effective characteristic of the shearing experiment is similar both for an elastic matrix with rigid inclusions and for a viscous fluid, see, for example^20^. Note that application of the method (“trick”) of Padé-approximants works well for the investigation of suspensions. The expression for the effective viscosity of suspensions accurate to the first order of the concentration of hard inclusions was obtained by Einstein^27^. In the work^28^ (see also^20,26,28–30^, an expression for the effective viscosity, which is accurate up to the square of the concentration, was obtained. The experimental data show the divergence of the effective viscosity as the concentration approaches the threshold. Such a divergence, however, cannot be derived from any power-law expansion. In the simplest form of the PA method, a power series in the concentration of inclusions, for example, a series of shear modulus or effective viscosity of a suspension at *p* ≪ 1 can be represented as the ratio of two polynomials. In this way, based on the Einstein’s expression for the effective viscosity proportional to 1 + 2.5*p*^27^ or a more accurate approximation 1 + 2.5*p* + 5*p*^2^ ^28^, the value of the percolation threshold according to Einstein was estimated as *p*_*c*_ = 1/2, or, when the second order of concentration is taken into account, as a more accurate value *p*_*c*_ = 2/5, which describes the experimental data well^20,26^.

To determine the critical exponent, it is necessary to apply one of the versions of the PA method. For estimation of the critical exponent, one can use the so-called D-log method of Padé approximants^25^.

## Renormalization of the critical exponent

In our case, the application of the method of Padé approximants significantly differs from the applications considered in^20^. We did not aim at finding the behavior of ${G}^{{\rm{e}}}(p)$, in particular its critical exponent *S*_0_ in (2). The numerical value of the critical index *S*_0_ in (2) will be assumed known from the literature^12,31^. The problem consists in determination of the influence of an external magnetic field on this critical index. Therefore, we seek the renormalization of the critical index *S*(*H*) for ${G}^{{\rm{e}}}(p,H)$ by a magnetic field *H*, where *S*(*H* = 0) = *S*_0_.

The value of the critical exponent *S*_0_ for the effective shear modulus for a “purely” elastic problem in the absence of magnetic field (*H* = 0) in the two-dimensional case was found in^30^ (see also^11–14^) and is equal in the two-dimensional case to

6 $${S}_{0}=\mathrm{1.3.}$$

Let us use the variation of method of Padé approximants, proposed in^21^, specifically for the determination of the critical exponent. We seek the effect of magnetic field *H* on the shear modulus ${G}^{{\rm{e}}}(p,H)$ in the form of

7 $${G}^{{\rm{e}}}(p,H)=\mu {(1-\frac{p}{{p}_{c}})}^{-S(H)},$$

where *p*_*c*_ is the given constant. The method of Padé approximants allows one to reproduce the critical behavior (7) and to estimate the parameter *S*(*H*) (which is the critical exponent) from the expansion of (4).

Expansion of the logarithmic derivative of (7) into the Taylor series gives

8 $$\frac{d}{dp}\,{\rm{l}}{\rm{n}}\,{G}^{{\rm{e}}}(p,H)\approx \frac{S(H)}{{p}_{c}}{p}^{0}+...,$$

while the corresponding expansion of *G*^e^(*p*, *H*) from (4), taking into account given expression for ${G}^{{\rm{e}}}(p)$, yields

9 $$\frac{d}{dp}\,{\rm{l}}{\rm{n}}[{G}^{e}(p)+\mu \cdot p\cdot {\rm{\Omega }}(H)]\approx (\frac{{S}_{0}}{{p}_{c}}+{\rm{\Omega }}(H)){p}^{0}+...,$$

where ${G}^{{\rm{e}}}(p)$ is chosen accordingly to (2).

Comparing (8) and (9), we obtain the critical exponent of an effective elastic modulus renormalized by an external magnetic field:

10 $$S(H)={S}_{0}+{\rm{\Omega }}(H){p}_{c}.$$

Thus, the term dependent on external magnetic field is no longer linearly proportional to *p* as in (4), but it enters the power exponent in (7).

For a more accurate determination of the critical exponent, one can also use the Padé approximant^24^ for the function

${F}_{2}(p)=({p}_{c}-p)\frac{d}{dp}\,\mathrm{ln}\,f(p)$ in the point *p* = *p*_*c*_, where *f*(*p*) is a series expansion of ${\mu }^{-1}({G}^{{\rm{e}}}(p)+\mu \cdot p\cdot {\rm{\Omega }}(H))$ with respect to concentration *p*.

The Padé polynomial for the function *F*_2_(*p*) is chosen in the form:

11 $$P(p)=({S}_{0}+{\rm{\Omega }}(H){p}_{c})\frac{1+Ap}{1+Bp},$$

where *A* and *B* are the coefficients found by using the expansion for *f*(*p*) in the form:

12 $$f(p)=1+(\frac{{S}_{0}}{{p}_{c}}+{\rm{\Omega }}(H))p+\frac{{S}_{0}({S}_{0}+1)}{2{{p}_{c}}^{2}}{p}^{2}.$$

Now with the help of (11) we obtain the expression for the critical index:

13 $$S(H)=P({p}_{c})=\frac{(1+{S}_{0})({S}_{0}^{2}(2+{S}_{0})-2{S}_{0}^{2}{\rm{\Omega }}(H){p}_{c}-{S}_{0}{{\rm{\Omega }}}^{2}(H){p}_{c}^{2})}{{S}_{0}(1+{S}_{0})(2+{S}_{0})-{\rm{\Omega }}(H){p}_{c}(1+{S}_{0})(2+3{S}_{0})-2{{\rm{\Omega }}}^{2}(H){p}_{c}^{2}(2+3{S}_{0})-2{{\rm{\Omega }}}^{3}(H){p}_{c}^{3}}.$$

For Ω (*H*)*p*_*c*_ < *S*_0_, Equation (13) gives (10), and it satisfies the equality *S*(*H* = 0) = *S*_0_.

The details of derivation of Eq. (13) are provided in the Appendix 1.

MAEs are usually characterized by the magnitude of the relative magnetorheological effect (MRE), which is defined as the relative change of the shear modulus due to applied magnetic field:

14 $$MRE=\frac{{G}^{{\rm{e}}}(H\ne 0)-{G}^{{\rm{e}}}(H=0)}{{G}^{{\rm{e}}}(H=0)}.$$

**Figure 3:**
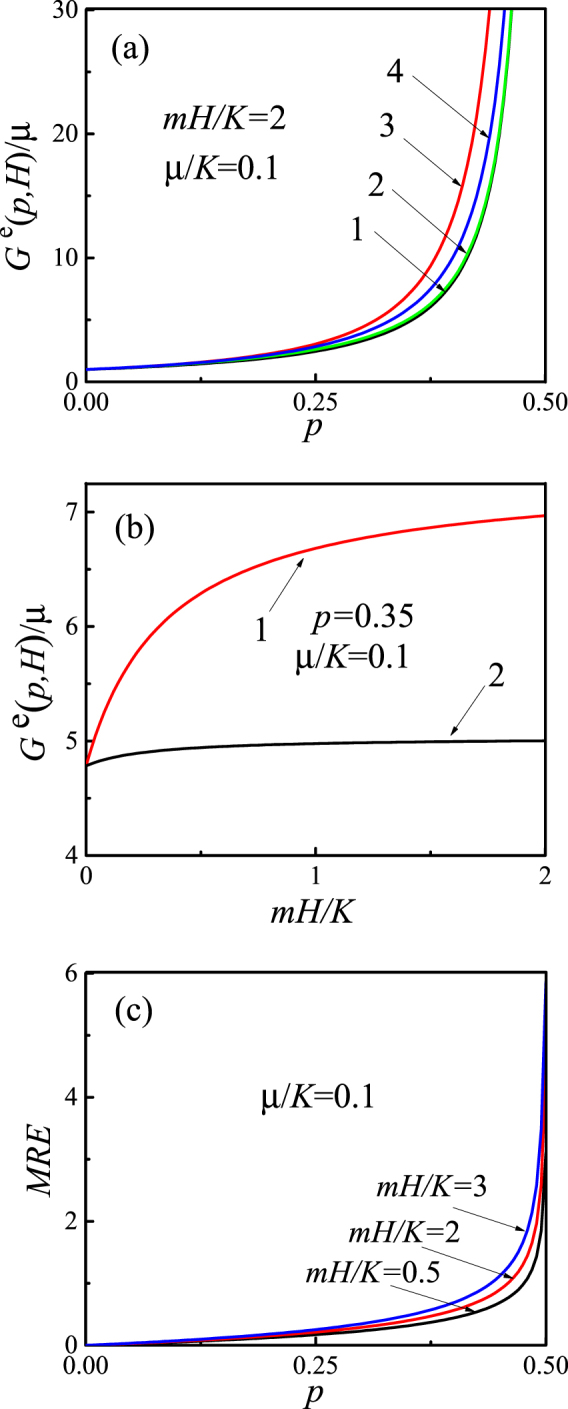
Field and concentration dependences of the normalized effective shear modulus for *μ*/*K* = 0.1. (**a**) Dependences of the shear modulus on the concentration *p* in the field *Hm*/*K* = 2 for different models: the black curve 1 is calculated from Eq. (2), the green curve 2 is obtained from Eq. (4), the red curve 3 takes into account the renormalization and it is calculated from Eq. (7) with exponent (10), the blue curve (4) takes into account the self-consistent approach and it is obtained from Eq. (15); (**b**) Dependence of the normalized shear modulus on applied magnetic field; (**c**) Dependence of the relative magnetorheological effect on the filler concentration in the framework of the renormalized percolation model for different external fields *mH*/*K* = 0.5, 2, 3.

Figure 3 shows the concentration and field dependences of the effective shear modulus. As can be seen from Fig. 3a, the effective modulus with a renormalized critical index increases with increasing concentration much faster than the module without normalizing the modulus. Figure 3b shows the growth of the effective modulus with the external magnetic field. It is seen that the effective shear modulus nonlinearly depends on external magnetic field and goes to saturation in large magnetic fields. Fig. 3с presents the dependence of the relative MR effect on the concentration of inclusions *p*. It is seen that the relative MR effect significantly grows with increasing concentration of inclusions *p*. More precisely, it theoretically goes to infinity when *p* → *p*_*c*_. This is the consequence of the dependence of the critical index on the external magnetic field and the approximations made (e.g. infinitely rigid inclusions). The obtained expression ((7) together with (10)) is asymptotic and is valid in the region *p* < *p*_c_. Similar to the theory of “smeared” phase transitions, it has to be expected that the MRE will take large (up to 10^6^%) but finite values in the vicinity of *p*_*c*_, as it was indeed observed in experiments^9^.

## Renormalization in a self-consistent model

Let us go beyond the model (2), applying a self-consistent approach, which allows one to consider the case of higher concentrations. We examine one more variant of the linear approximation (3). Renormalization of the critical index by magnetic field (10) was performed on the basis of expression (4) for ${G}^{{\rm{e}}}(p,H)$. This expression has been obtained in^19^ in the approximation of a single inclusion with infinite large elastic moduli in an elastic medium with the shear modulus *μ*. Several approaches are known allowing one to improve calculation of effective coefficients based on the approximation of single inclusions using the self-consistency method. The most successful approach is the Bruggeman-Landauer approximation^12,14,15^. In this approximation, it is assumed that the solitary inclusion of the first phase is not surrounded by the first phase but by the medium with the effective elastic moduli to be found. The same assumption is also valid for the first phase.

Similar (but not exactly the same) approach can be used during derivation of Eq. (4). First, one may assume that the solitary inclusion is immersed into a medium with effective elastic moduli of the composite materials, calculated in the absence of magnetic field. In this case, Eq. (3) should be replaced by

15 $${G}_{SC}^{{\rm{e}}}(p,H)={G}^{e}(p)+{G}^{e}(p)\cdot p\cdot {\rm{\Omega }}(H).$$

The expressions for the effective shear modulus ${G}_{SC}^{{\rm{e}}}(p,H)$ will be different from (3). The detailed analysis of ${G}_{SC}^{{\rm{e}}}(p,H)$ and some other modifications of (3) is not the subject of this paper and will be given in the separate publication.

Here we are interested in the self-consistent approach, which instead of (4) leads to expression (15) for the shear modulus. The dependence of the shear modulus, obtained from (15) in Fig. 3(a) is given by curve 4. This curve 4 goes much higher than curves 1 and 2 obtained with the help of expressions (2) and (4), and it is closer to curve 3 obtained by means of renormalization. However, as it is easily seen from (15), self-consistency does not lead to the effect of the anomalous growth of MRE when the concentration *p* tends to the threshold value *p*_c_ (*p* → *p*_*c*_).

The most interesting question is how the self-consistency leading to ${G}_{SC}^{{\rm{e}}}(p,H)$ influences the critical exponent *S*(*H*). For this calculation, it is necessary to substitute into (9) the expression for ${G}_{SC}^{{\rm{e}}}(p,H)$ instead of ${G}^{e}(p)+\mu \cdot {\rm{\Omega }}(H)p$. The first term in the expansion of the logarithmic derivative gives the expression for the renormalized critical exponent *S*(*H*). It turns out that the critical exponent for ${G}_{SC}^{{\rm{e}}}(p,H)$ remains the same as for ${G}^{e}(p,H)$ and does not depends on self-consistency. One can speak about “universality” of the renormalized critical index *S*(*H*) = *S*_0_ + Ω(*H*)*p*_*c*_.

## Discussion

There are macroscopically inhomogeneous media (composite materials) where an analog of phase transition of the second order is observed. This is the percolation transition. Examples are elastic two-phase materials with a strong difference in the elastic properties of the phases. Percolation transitions (they mean sharp change of the effective properties of the entire system, i.e. the composite material as a whole) are observed near the percolation threshold, when the concentration of one of the phases approaches the percolation threshold. However, often such macroscopically inhomogeneous media exist and are considered at low concentrations *p* ≪ 1. This case is also interesting for investigation and has broad field of applications. For example, in the pioneering work of Albert Einstein the viscosity of a suspension (solid particles in a viscous liquid), first considered in detail with accuracy of the first order *p*^1^. Later, refined theories were developed with accuracy up to *p*^2^ and further. It is quite clear that a theory developed up to an arbitrarily large order of *p* (if it is possible) does not describe a percolation transition specifying divergence when approaching the percolation threshold ~(*p*_*c*_ − *p*)^−*S*^. The same thing happens in phase transitions. Expansion of the order parameter in powers of temperature does not give its critical behavior near the phase transition temperature. Nevertheless, having only such approximations and using the method of Padé approximants it is possible to re-establish the critical behavior of the order parameter and with good approximation to calculate the basic “world” constants of the theory, i.e. critical indices. In this case, as originally suggested, the expansion of the logarithmic derivative of the order parameter is used. The divergence of the shear modulus at the percolation threshold is explained by the approximation of infinitely rigid particles. Such “hardened” states with touching particles and therefore diverging elastic modulus are well known in the literature^32^. In real-word applications, the effective modulus will be limited by the large but finite modulus of ferromagnetic inclusions.

## Conclusion

In the present work, the same approach as in the theory of second order phase transitions has been applied. However, we did not use it to find the critical exponent of effective shear modulus in the absence of magnetic field, which we considered to be known. We employed it to determine the renormalization of this exponent by a magnetic field. As it turned out, the critical index describing the elastic behavior of an MAE material in a magnetic field near the percolation threshold depends on the magnetic field. That allows one to describe the dependences of the absolute and relative magnetorheological effect in magnetoactive elastomers on external magnetic field and concentration of soft magnetic inclusions. In particular, the relative magnetorheological effect should strongly increase when the concentration of filler particles approaches the percolation threshold (*p* < *p*_*c*_, *p* → *p*_*c*_). Our renormalization approach can be applied to many other materials, also in the 3D case. For example, an extension to magnetorheological fluids and gels^30,33^ is obvious.

## Electronic supplementary material

Appendix 1
