# Supplementary material for: Renormalization of the critical exponent for the shear modulus of magnetoactive elastomers
Source: Sci Rep. 2018 Mar 13;8:4397. doi: 10.1038/s41598-018-22333-6 (PMC5849735; doi:10.1038/s41598-018-22333-6)
Supplement: Supplementary file 1 — Appendix 1 [file 41598_2018_22333_MOESM1_ESM.pdf]

Renormalization of the critical exponent for the shear modulus of magnetoactive elastomers

Andrei A. Snarskii[1,2], Viktor M. Kalita[1,3], Mikhail Shamonin[4,*]

[1] *Igor Sikorsky Kyiv Polytechnic Institute, Prospekt Peremohy 37, 03056 Kiev, Ukraine*
[2] *Institute for Information Recording NAS of Ukraine, Shpaka Street 2, 03113 Kiev, Ukraine*
[3] *Institute of Physics NAS of Ukraine, Prospekt Nauky 46, 03028 Kiev, Ukraine*
[4] *East Bavarian Centre for Intelligent Materials (EBACIM), Ostbayerische Technische Hochschule Regensburg, Prüfeninger Strasse 58, 93049 Regensburg, Germany*

Appendix 1.

Calculating $F_2(p)$ and expanding it into a series we find

$$F_2(p) \approx \left(S_0 + \Omega(H)p_c\right)(1 - \frac{\Omega(H)\left(1+\Omega(H)p_c + 2S_0\right)}{S_0 + \Omega(H)p_c}p + \\ + \frac{2p_c{}^3\Omega^3(H) + 2p_c{}^2\Omega^2(H)\left(1+3S_0\right) - \left(2S_0 + 3S_0{}^2 + S_0{}^3\right)}{2p_c{}^2\left(S_0 + \Omega(H)p_c\right)}p^2) \quad . \tag{16}$$

The Padé polynomial (and thereby its expansion into a series with respect to the concentration *p*) is chosen in the following form

$$P(p) = \left(S_0 + \Omega(H)p_c\right)\frac{1+Ap}{1+Bp} \approx \left(S_0 + \Omega(H)p_c\right)\left[1 + (A-B)p + B(B-A)p^2\right] \ . \tag{17}$$

Comparing the coefficients of the expansion of $F_2(p)$ and $P(p)$ we find the coefficients *A* and *B*

$$A = -\frac{S_0^2\left(1+S_0\right)\left(2+S_0\right) + 2S_0\Omega(H)p_c\left(S_0 - S_0^2 + 1\right) + \Omega^2(H)p_c^2\left(5S_0 - S_0^2 + 2\right) + 2\Omega^3(H)p_c^3}{2\Omega(H)p_c^2S_0\left(1+2S_0\right) + 2\Omega^2(H)p_c^3\left(1+3S_0\right) + 2\Omega^3(H)p_c^4} \ , \tag{18}$$

$$B = \frac{2\Omega^3(H)p_c^3 + 2\Omega^2(H)p_c^2\left(1+3S_0\right) + \Omega(H)p_cS_0\left(1+3S_0\right) - S_0\left(1+S_0\right)\left(2+S_0\right)}{2\Omega(H)p_c^2\left(2S_0 + \Omega(H)p_c + 1\right)} \quad . \tag{19}$$

Finally, we substitute them into the Padé polynomial, $P(p) = \left(S_0 + \Omega(H)p_c\right)\left(1+Ap\right)/\left(1+Bp\right)$, and find the critical exponent, which is equal to the Padé polynomial at $p = p_c$. That is Equation (13), where, as it should be, $S(H=0) = S_0$.

[*] Corresponding author. E-Mail: mikhail.chamonine@oth-regensburg.de

Expanding $S\left(H\right)$ into power series with respect to the additional magnetic term $\Omega\left(H\right)$, we get in the first approximation, as it should be, $S\left(H\right)=S_0+\Omega\left(H\right)p_c$, obtained earlier in the simplest approximation (10).
